# Competitive Endogenous RNA Network Involving Immune Subgroups, Infiltration, and lncRNAs in Prostate Cancer

**DOI:** 10.3390/genes16050527

**Published:** 2025-04-29

**Authors:** Wenkang Niu, Tingting Zhang, Lei Ma

**Affiliations:** 1 College of Life Science, Shihezi University, Shihezi 832000, China; wenkang_niu@163.com; 2 Key Laboratory of Oasis Town and Mountain-Basin System Ecology of Bingtuan, Shihezi University, Shihezi 832000, China

**Keywords:** prostate cancer, ssGSEA, immune infiltration, WGCNA, ceRNA

## Abstract

Prostate cancer (PCa) is the most frequently diagnosed malignancy in the male genitourinary tract. However, the regulatory mechanism of competitive endogenous RNAs (ceRNAs) in PCa remains unclear. In this study, we first performed immune scores of mRNA data from 481 PCa samples using single-sample Gene Set Enrichment Analysis (ssGSEA). Based on the immune scores, we then evaluated the tumor immune microenvironment and analyzed 28 types of immune cells in PCa, we constructed a comprehensive network with four lncRNAs (MEG3, PCAT1, SNHG19, TRG-AS1), three miRNAs (hsa-miR-488-3p, hsa-miR-210-5p, hsa-miR-137), and twenty-seven mRNAs (including H2AFJ, THBS1, HPGD). Among the 28 immune cell types, seven immune cell types were found to be significantly associated with clinical characteristics. These network nodes have prognostic significance in multiple cancers and play critical roles in malignancy development, indicating the network’s predictive capability. We also observed a strong correlation (r = 0.6) between T-helper type 1 (Th1) cells and lncRNA network modules. The network connectivity highlights the association between immune therapy biomarkers for PCa, particularly those related to H2AFJ, THBS1, and HPGD. These findings provide valuable insights into the ceRNA regulatory network and its implications for immune-based therapies in PCa.

## 1. Introduction

Prostate cancer (PCa) is the most common malignant tumor of the male genitourinary system [1]. It is frequently diagnosed in hundreds of countries and ranks as the second-leading cause of cancer-related deaths in males. In 2020, PCa accounted for an estimated 1.414 million new cases worldwide and led to around 375,000 deaths [2]. The development of serum prostate-specific antigen (PSA) testing has significantly improved the detection rate of PCa [3]. However, due to the high heterogeneity of PCa, clinical challenges such as treatment resistance and tumor recurrence continue to rise [4].

Within cells, a competitive endogenous RNA (ceRNA) mechanism exists, wherein various molecules such as mRNA, lncRNA, and pseudogenes can regulate each other’s expression levels by competitively binding to the same microRNA response elements (MREs) and interacting with corresponding miRNAs [5]. The involvement of ceRNA networks in cancer progression has attracted considerable interest in recent studies. For example, in a ceRNA network focused on the prostate cancer biomarker UBASH3B (Ubiquitin-associated and SH3 domain-containing B), UBASH3B directly engages with the immune-related gene LCP2, playing a role in mediating inflammatory responses [6]. Moreover, UBASH3B can bind to miR-148a-3p, negatively regulating platelet activation mediated by FcγRIIA [7]. Numerous studies have investigated the interactions among miRNAs, mRNAs, and lncRNAs through ceRNA networks to elucidate their impact on cancer. In PCa, for example, miR-17-5p inhibits the expression of tissue inhibitor of metalloproteinase 3 (TIMP3) in PCa and is involved in the expression of EIF3H, HELLS, and DNAL1 genes within the same network, wherein the lncRNA URS000048C392 plays a regulatory role [8]. Thus, enhancing our understanding of ceRNA-based regulatory mechanisms will contribute to a deeper comprehension of the pathogenesis of PCa.

The aim of the present study was to utilize the ssGSEA algorithm for calculating immune scores and stratify PCa samples into high and low ssGSEA score groups based on their immune scores. We then assessed the disparities in the infiltration levels of 28 immune cells between these two groups. To identify lncRNAs associated with immune cells, we performed differential expression analysis of these lncRNAs between the high and low ssGSEA score groups. Subsequently, we constructed a ceRNA network by integrating these differentially expressed lncRNAs with the differentially expressed miRNAs and mRNAs. The nodes within this network were identified as potential prognostic markers for PCa patients. These findings offer new insights into personalized treatment strategies for PCa and provide potential prognostic targets for improving the survival outcomes of PCa patients.

## 2. Materials and Methods

### 2.1. Data Download

The RNA-seq data of PCa patients were downloaded from the TCGA database (https://www.cancer.gov/) (accessed on 30 March 2023), and we excluded the normal samples, resulting in 481 remaining cancer samples. The miRNA data were sourced from the UCSC Xena database (https://xenabrowser.net/datapages/) (accessed on 30 March 2023). For lncRNA data, the gtf file downloaded from the Ensembl database (https://ftp.ensembl.org/pub/release-109/gtf/homo_sapiens/) (accessed on 22 February 2023) was utilized, with the data version specified as genebuild-last-updated 2019-06. We downloaded clinical data for 500 PCa cases from the cBioPortal database (http://www.cbioportal.org/) (accessed on 22 February 2023) (data version: 2023-03). Clinical data on Gleason scores for 500 cases were obtained using the R package “TCGAbiolinks” (data version: 2023-03). To ensure data consistency, we used the inner_join function from the R package “dplyr” to integrate clinical and transcriptomic data based on RNA-seq sample IDs. As a result, we obtained a final dataset of 481 samples with complete clinical and transcriptomic information.

### 2.2. Immunological Grouping and Its Validation

In this study, RNA-seq expression data (FPKM format) from 481 prostate cancer samples were obtained from the TCGA database and converted into TPM (Transcripts Per Million) values. Lowly expressed genes with an average expression of zero were removed, and the avereps function was used to calculate the median expression for duplicate genes, resulting in a standardized expression matrix. Based on 28 immune-related gene sets from MSigDB database, immune scores for each sample were calculated using the ssGSEA method implemented in the R package “GSVA” (parameters: GSVA::ssgseaParam(geneSets = geneSet, alpha = 0.25, normalize = TRUE)). Euclidean distance was calculated from the immune scores, followed by hierarchical clustering using the hclust function with the “ward.D2” method. To determine the optimal number of clusters, Silhouette analysis was performed, revealing that k = 2 yielded the highest average silhouette width (0.33). Based on the median immune scores of each cluster (Cluster 1: 0.462; Cluster 2: 0.524), Cluster 2 was defined as the high immune activity group (high ssGSEA score group, *n* = 156), and Cluster 1 as the low immune activity group (low ssGSEA score group, *n* = 325). To further evaluate the clustering performance, t-Distributed Stochastic Neighbor Embedding (t-SNE) analysis was conducted using the R package “Rtsne” [9] (parameters: perplexity = 10, max_iter = 500) to visualize the distribution of samples in a two-dimensional space.

To confirm the immune classification derived from ssGSEA scores, the expression profiles of Programmed Death-Ligand 1 (PD-L1) and Human Leukocyte Antigen (HLA) family genes were analyzed, as these genes play key roles in tumor immune escape mechanisms [10]. The expression level of PD-L1 was determined by measuring the expression level of the CD274 gene. Additionally, the expression levels of 22 HLA family genes were extracted from the mRNA expression data for further analysis. To ensure data comparability and stability, all gene expression values were transformed using log_2_(FPKM + 1) prior to analysis.

### 2.3. Tumor Microenvironment

To validate the immune grouping based on ssGSEA scores, the R package “ESTIMATE” was employed. This package utilizes estimation algorithms that leverage RNA-seq expression levels to calculate the levels of immune cell and stromal cell infiltration in the tumor microenvironment. By applying this method, we were able to calculate various scores for the PCa samples, including tumor purity, estimation score, immune score, and stromal score. These scores provided further validation of the effectiveness of the ssGSEA grouping. To visualize the results, a clustering heatmap was generated. Additionally, box plots depicting the tumor purity, estimation score, immune score, and stromal score in the two immune groups were created using the R package “ggplot2”.

### 2.4. Assessment of Immune Cell Infiltration

The relative abundance of immune cells was estimated based on the ssGSEA method, and the analysis data came from the ssGSEA result file, ssgseaOut.txt (Supplementary Table S4). The gene sets for the 28 immune cell types were referenced from the TISIDB database (http://cis.hku.hk/TISIDB/data/download/CellReports.txt) (accessed on 21 April 2023) (Supplementary Table S5). After dividing the samples into high and low immune groups based on immune scores, we used the Wilcoxon rank-sum test (wilcox.test) to assess differences in immune cell infiltration levels between the two groups. To control the false discovery rate (FDR), all *p*-values were corrected using the Benjamini–Hochberg method, and significance was determined based on the FDR-adjusted *p*-value (adjusted *p* < 0.05). Immune cells showing significant differences were then analyzed for their correlation with clinical characteristics. For clinical data selection, we chose complete data for T stage, N stage, Gleason score, and age (AGE), with Pearson’s correlation test used for AGE and the Wilcoxon test used for the other clinical features.

### 2.5. Construction of a Weighted Gene Co-Expression Network

We filtered out 16,908 lncRNA genes from the gtf file and obtained FPKM expression data for 3859 lncRNAs. These lncRNAs were merged and utilized for further analysis to predict their potential roles in PCa progression. The expression data of these 3859 lncRNAs were analyzed using Weighted Gene Co-expression Network Analysis. Initially, Pearson correlation was used to assess the weighted co-expression relationships among the participants in the adjacency matrix of all datasets. The matrix was then transformed into a Topological Overlap Matrix (TOM) using the TOM similarity function. Subsequently, a dynamic tree-cutting method was applied to cluster at least 30 co-expressed genes into different modules. The modules with the highest and lowest module-trait correlations were selected for further analysis. Within these two modules, differential expression analysis was conducted using the R package “DESeq2” to identify lncRNAs associated with differentially expressed immune cells.

### 2.6. Construction of ceRNA Network

The R package “DESeq2” (|logFC| > 1, *p* < 0.05) was used to perform differential expression analysis on RNAs between high and low ssGSEA score groups, and volcano plots were generated. The differentially expressed RNAs were then used for subsequent database searches. After predicting miRNAs using the miRcode database (http://www.mircode.org/index.php) (accessed on 30 March 2023) and the ENCORI database (https://starbase.sysu.edu.cn/) (accessed on 1 January 2023) (data version: 2023-01), we obtained miRNAs that interacted with lncRNAs and established lncRNA-miRNA pairs. We predicted the target genes of miRNAs using the miRTarBase database (https://www.targetscan.org/vert_80/) (accessed on 30 March 2023) and the ENCORI database, and identified overlapping target genes between these two databases to obtain miRNA-mRNA pairs. Finally, the obtained pairs were imported into Cytoscape software (version 3.9.1) to construct the ceRNA network.

### 2.7. Functional Enrichment Analysis

To explore the biological relevance of the mRNAs, functional enrichment analysis was conducted on both differentially expressed mRNAs and those within the ceRNA network via the DAVID database (https://david.ncifcrf.gov/) (accessed on 30 May 2023). Gene Ontology (GO) terms with significant enrichment (*p* < 0.05) were visualized as bar plots using the R package “ggplot2”.

### 2.8. Statistical Analysis

All statistical analyses were carried out using R software (version 4.1.0, http://www.R-project.org) (accessed on 5 March 2023). A two-tailed *p*-value below 0.05 was deemed statistically significant. The Wilcoxon test was applied to compare high and low ssGSEA score groups in PCa, while Spearman correlation analysis was used to explore associations between lncRNAs and differentially expressed immune cell types. In the statistical methods section, throughout the manuscript, the notations *, **, ***, and **** denote *p* < 0.05, 0.01, 0.001, and 0.0001, respectively, while “ns” indicates *p* > 0.05.

## 3. Results

### 3.1. Immune Grouping

**Figure 1:**
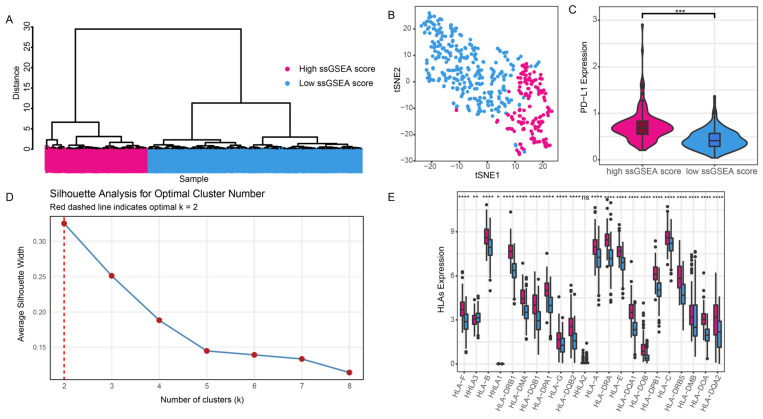
Immune grouping. (**A**) Clustering of PCa samples based on ssGSEA immune scores. (**B**) t –SNE analysis of high and low ssGSEA score groups. (**C**) Expression of PD–L1 in high and low ssGSEA score groups. (**D**) Expression of HLAs in high and low ssGSEA score groups. In the box plot, red represents the group with a high ssGSEA score, and blue represents the group with a low ssGSEA score. (**E**) Silhouette analysis for optimal cluster number. Red dashed line indicates the optimal number of clusters (k = 2). The notations *, **, ***, and **** denote *p* < 0.05, 0.01, 0.001, and 0.0001, respectively, while “ns” indicates *p* > 0.05.

To obtain immune subgroups, we calculated immune scores using ssGSEA. Silhouette analysis was performed to assess the clustering effectiveness for different values of k. The results showed that k = 2 yielded the highest average silhouette width (0.33), indicating that the binary clustering approach provided the optimal clustering result (Figure 1E). Based on this, the 481 PCa samples were divided into a high ssGSEA score group (*n* = 156) and a low ssGSEA score group (*n* = 325). Hierarchical clustering revealed a clear division of samples into two distinct groups (Figure 1A). Additionally, t-SNE dimensionality reduction demonstrated a clear separation between the high and low score groups (Figure 1B).

To assess the differences between the immune groups in PCa, we examined the expression levels of HLAs and PD-L1, which are often used as biomarkers in tumor immune evasion mechanisms. Our results revealed significant differences in the expression levels of HLAs and PD-L1 between the high and low ssGSEA score groups (Figure 1C,D). Specifically, the high ssGSEA score group exhibited higher expression levels of HLAs and PD-L1 compared to the low ssGSEA score group. These results reflect the validity of our grouping.

### 3.2. Differences in Immune Grouping in the Tumor Microenvironment

**Figure 2:**
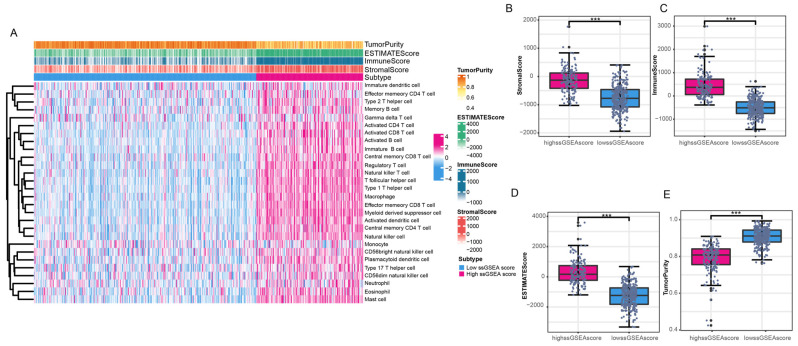
Validation of immune grouping in the tumor microenvironment. (**A**) Infiltration levels of 28 immune-related cells in high and low ssGSEA score groups, combined with grouping information showing immune score, stromal score, tumor purity, and estimate score of patients. (**B**–**E**) Relationships between high and low ssGSEA score groups and stromal score, immune score, estimate score, and tumor purity. The notations *** denote *p* < 0.001.

To assess the differences in immune grouping within the tumor microenvironment, we analyzed tumor microenvironment indicators in PCa based on the immune grouping. Tumor purity, stromal score, immune score, and estimate score were calculated for 481 PCa samples (excluding normal samples) based on the immune grouping. In the high ssGSEA score group, the stromal score, immune score, and estimate score were higher compared to the low ssGSEA score group, while the tumor purity showed the opposite trend (Figure 2A). These findings suggest that the high ssGSEA score group has elevated levels of infiltrating stromal cells and immune cells, but lower tumor purity. The differences in tumor purity, estimate score, immune score, and stromal score between the high and low ssGSEA score groups were statistically significant (Figure 2B–E). Hence, the notable disparities in various tumor microenvironment indicators between the high and low ssGSEA score groups provide further validation of the effectiveness of our immune grouping.

### 3.3. Infiltration Analysis of Immune Cells

**Figure 3:**
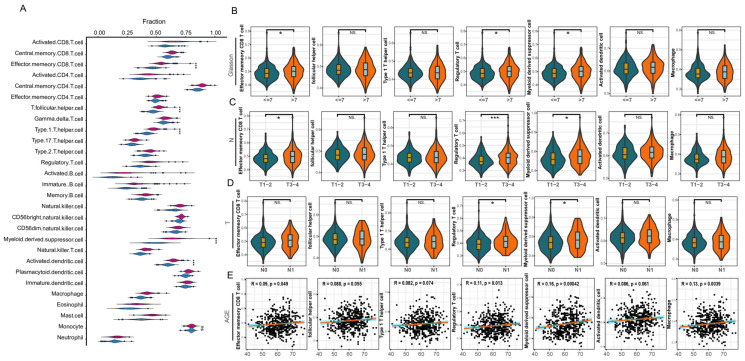
Immune infiltration. (**A**) Violin plot showing the infiltration levels of 28 immune cells in the high and low ssGSEA score groups. (**B**–**D**) Relationships between significantly different immune cells and clinical features (N, T stage, and Gleason grade). (**E**) Relationships between significantly different immune cells and the clinical feature AGE. The red line indicates the linear regression fit, and the blue shaded area represents the 95% confidence interval. The notations * and *** denote *p* < 0.05, and 0.001, respectively, while “NS” indicates *p* > 0.05.

To evaluate immune cell infiltration in PCa, we measured the infiltration levels of 28 immune cell types across 481 PCa samples. Significant differences were observed in the infiltration levels of the 28 immune cells between the high ssGSEA score group and the low ssGSEA score group (Figure 3A). Among these, seven immune cells exhibited the most pronounced differences (*p* < 0.001): Effector memory CD8^+^ T cells, follicular helper T cells (Tfhs), Type 1 T helper cells (Th1s), regulatory T cells (Tregs), myeloid derived suppressor cells (MDSCs), activated dendritic cells (DCs), and Macrophages. We further evaluated the behavior of these seven immune cells in the tumor microenvironment (Supplementary Figure S1). They displayed positive correlations with stromal score, immune score, and estimate score, while exhibiting negative correlations with tumor purity.

Next, we stratified patients based on AGE, Gleason score, and TNM stage, and explored the correlations between these seven immune cells and the clinical features of PCa (excluding patients with incomplete TNM stage). The performance of different immune cells varied across different clinical features (Figure 3B–E). For instance, Effector memory CD8+ T cells exhibited significant differences across different T stages and Gleason scores; Tregs showed significant differences across different T stages, N stages, and Gleason scores; MDSCs displayed significant differences across different T stages, N stages, and Gleason scores; and Macrophages exhibited significant differences across different T stages and Gleason scores. Additionally, Effector memory CD8^+^ T cells, Tregs, MDSCs, and Macrophages all showed a weak positive correlation with AGE. These results indicate that the infiltration levels of these seven immune cells differ significantly based on various clinical features of PCa. Moreover, the extent of immune cell infiltration increased with higher Gleason scores, T stages, N stages, and AGE in PCa. Therefore, these immune cells play crucial roles in the progression of PCa and contribute to the tumor microenvironment.

### 3.4. lncRNAs Associated with the Seven Immune Cells

**Figure 4:**
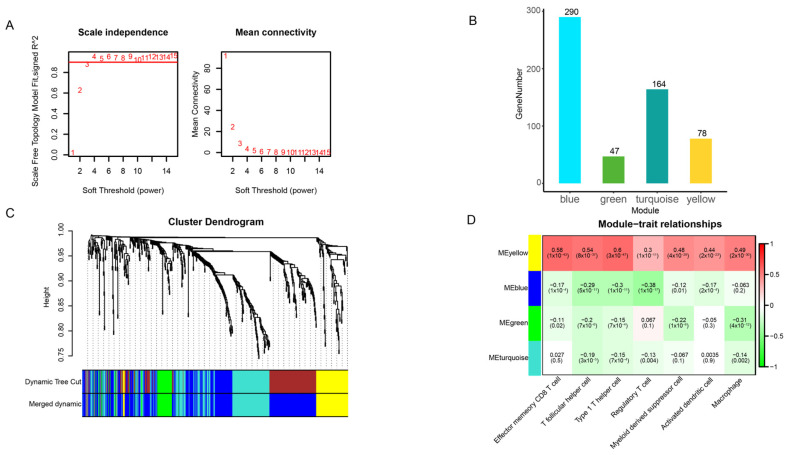
Seven immune-cell-related lncRNAs. (**A**) Analysis of the network topology structure of lncRNA transcriptome data at different soft-threshold values. (**B**) Histogram showing the number of genes in each module. (**C**) Gene clustering tree and module segmentation. Different colors represent different gene modules respectively, where genes within a module share similar expression patterns and may be functionally related. (**D**) Heatmap showing the correlation between seven significantly different immune cells and modules.

To identify lncRNAs associated with the seven immune cells, we employed the WGCNA tool (version 1.73). Initially, we filtered the lncRNA expression data of PCa and retained 3859 lncRNAs with high expression by excluding low expression data. To construct a scale-free network, we selected a weight value β = 5, at which the R^2^ (scale-free topology model fit, signed R^2^) initially reached 0.90 (Figure 4A). By merging modules, we obtained a total of four modules (Figure 4C). The number of lncRNAs within each module ranged from 47 to 290, determined by gene clustering based on expression correlation (Figure 4B).

To delve deeper into the modules associated with the seven immune cells and lncRNAs, we conducted a correlation analysis between the modules and immune cells. As a result, we identified modules that exhibited significant correlation with the immune cells (Figure 4D). Among these modules, the yellow and blue modules displayed the highest correlation with the seven immune cells. Consequently, we selected these two modules for further analysis. Ultimately, we obtained a total of 367 lncRNAs (after eliminating duplicates) that were associated with the seven immune cells, which served as the foundation for constructing the ceRNA network.

### 3.5. Construction of the ceRNA Network

**Figure 5:**
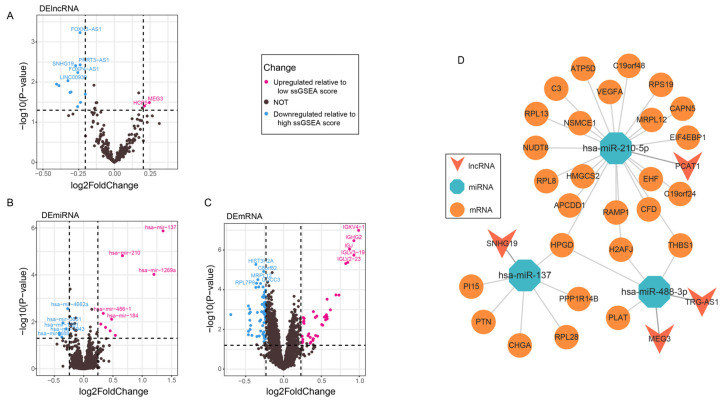
Construction of the ceRNA network. (**A**–**C**) Volcano plots showing the differential expression of lncRNAs, miRNAs, and mRNAs (pink indicates upregulated relative to low ssGSEA score, blue indicates downregulated relative to high ssGSEA score). In the volcano plot, the horizontal dashed line represents the statistical significance threshold, and the vertical dashed line indicates the fold change threshold. (**D**) ceRNA network (blue represents miRNA, red represents lncRNA, orange represents mRNA).

To explore the interplay between miRNAs, mRNAs, and lncRNAs in PCa, we conducted differential expression analysis on RNAs based on the high and low ssGSEA score groups. This analysis allowed us to identify differentially expressed lncRNAs, miRNAs, and mRNAs, which were then used to construct a ceRNA regulatory network. Notably, we observed significant expression differences for all types of RNAs between the high and low ssGSEA score groups (Figure 5A–C). Specifically, 12 lncRNAs were upregulated, 2 lncRNAs were downregulated (Supplementary Table S3), 9 miRNAs were upregulated, 6 miRNAs were downregulated (Supplementary Table S2), 41 mRNAs were upregulated, and 67 mRNAs were downregulated (Supplementary Table S1).

Using the differentially expressed RNAs, we established lncRNA-miRNA and miRNA-mRNA pairs through database matching, resulting in the construction of a ceRNA network associated with the ssGSEA score group in PCa (Figure 5D). This network comprises 4 lncRNAs, 3 miRNAs, and 27 mRNAs. Notably, HPGD, H2AFJ, and THBS1 display higher connectivity within the network and are regarded as hub genes.

### 3.6. Binding of Seed Regions Between mRNAs, miRNAs, and lncRNAs in PCa

Based on our analysis, we identified three mRNAs (HPGD, H2AFJ, and THBS1) that pair with three miRNAs (hsa-miR-122-5p, hsa-mir-488-3p, and hsa-mir-137) in the ceRNA network. These interactions are characterized by seed region binding types such as 7mer-A1, 7mer-m8, 8mer, and 6mer, as described in Table 1. Additionally, hsa-mir-488-3p and hsa-mir-137 were found to pair with three lncRNAs (MEG3, TRG-AS1, and SNHG19) with seed region binding types of 7mer-m8 and 8mer, respectively, as shown in Table 2. It is worth noting that the seed regions of these miRNAs exhibit complete complementarity to the 3′UTR of the target mRNAs and lncRNAs, thereby enhancing the silencing effect of miRNAs on their targets. This highlights the potential therapeutic significance of studying miRNA-mediated targeting of mRNAs in PCa, particularly focusing on the high specificity of the identified miRNAs.

### 3.7. Functional Enrichment of Hub Genes

**Figure 6:**
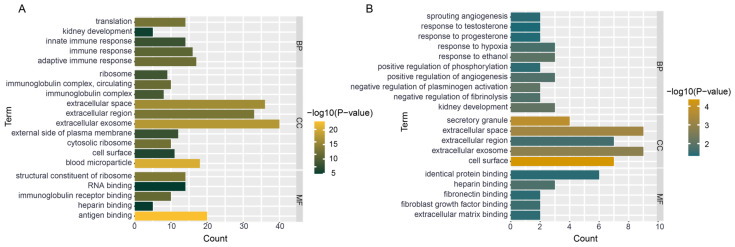
Gene enrichment analysis. (**A**) Bar plot of GO enrichment for 108 differentially expressed genes. (**B**) Bar plot of GO enrichment for 27 genes involved in the ceRNA network.

To understand the functions of the differentially expressed genes, we performed GO enrichment analysis on the 108 genes (Figure 6A). The enriched GO terms associated with these genes span various biological processes (BPs), such as immune response, as well as adaptive and innate immune responses. They are also implicated in diverse cellular components (CCs), including extracellular exosomes, extracellular spaces, and plasma membranes. Furthermore, these genes are involved in a range of molecular functions (MFs), such as ribosomal structural constituents, immunoglobulin receptor binding, heparin binding, antigen binding, and more.

Comparing the 27 genes in the ceRNA network to the previous set of 108 genes, we found similarities in the enriched CC and MF entries. However, there were notable differences in the enriched BP terms. Specifically, the BP entries for the ceRNA network genes included responses to testosterone and responses to progesterone, among others. These enrichment results indicate that the genes involved in the ceRNA network have crucial roles in the progression of PCa (Figure 6B).

## 4. Discussion

The present study aimed to elucidate the molecular mechanisms underlying the progression of PCa by constructing a ceRNA network, which consisted of 4 lncRNAs, 3 miRNAs, and 27 mRNAs, based on the grouping of PCa samples into high and low ssGSEA score groups. The ceRNA network provides valuable insights into the regulatory interactions among these RNA molecules. Notably, the seed regions of the lncRNAs and miRNAs, as well as the miRNAs and mRNAs in the network, exhibit complete complementarity, suggesting potential functional interactions.

Furthermore, we identified seven immune cell types (DCs, Tfhs, Th1s, Tregs, MDSCs, Effector memory CD8^+^ T cells, and Macrophages) that are associated with the expression levels of PCa lncRNAs. These immune cells have been recognized as prognostic indicators in various cancers and are known to play crucial regulatory roles in different cancer types [11,12,13,14,15,16,17]. Their functions encompass important aspects such as antigen presentation, pro-inflammatory response, immune regulation, and the maintenance of cellular homeostasis, among others.

By exploring the correlation between clinical features and these seven immune cells of PCa, we have uncovered new perspectives that hold promise for the treatment and management of PCa. This comprehensive investigation sheds light on the intricate interplay between immune cells, lncRNAs, and other RNA molecules, providing a foundation for further research and potential therapeutic interventions in PCa.

Several studies have previously constructed ceRNA networks in PCa using different approaches and datasets. For example, one study developed a ceRNA network comprising 14 lncRNAs, 31 miRNAs, and 162 mRNAs by conducting differential expression analysis between normal and PCa samples, employing a relatively lenient threshold [18]. Another study utilized the R package “GDCRNATools” to construct 61 ceRNA networks and selected the network with the strongest mRNA-lncRNA correlation. They further explored the role of mRNA CCNL2 in tumorigenesis by analyzing immune infiltration, drug sensitivity, and methylation within the ceRNA network [19]. In a different study, researchers focused on primary prostate cancer (pPCa) and metastatic prostate cancer (mPCa) samples to construct a distinct ceRNA network based on single-line regulation. This comprehensive network consisted of 267 miRNAs, 1280 lncRNAs, and 8392 mRNAs, enabling the identification of potential biomarkers through the study of miRNA-lncRNA-mRNA interactions and competition [20].

These studies collectively contribute to our understanding of the complex regulatory mechanisms involved in PCa through ceRNA networks. Each study has its unique characteristics, such as differentially expressed genes, selected tools, and a specific focus on different aspects of PCa. Our study, in comparison, provides valuable insights into the regulatory interactions within the ceRNA network by specifically considering the grouping based on ssGSEA scores. By investigating the unique characteristics of the high and low ssGSEA score groups, we aim to shed light on the molecular mechanisms associated with the immune microenvironment and its impact on PCa progression.

In comparison to these previously reported ceRNA networks, our network consists of a smaller number of nodes, reflecting the specific influence of the high and low ssGSEA score groups on the ceRNA regulatory network. By focusing on the grouping based on ssGSEA scores, we aimed to uncover distinct regulatory interactions potentially linked to the immune microenvironment, offering new insights into the molecular mechanisms driving PCa progression.

### 4.1. Immune Infiltrating Cells

Our analysis revealed seven immune cell types that exhibited significant differences in PCa: Effector memory CD8^+^ T cells, Tfhs, Th1s, Tregs, MDSCs, DCs, and Macrophages. These immune cells are known to play crucial regulatory roles in cancer and have been identified as prognostic indicators in various cancer types. Their functions encompass a wide range of activities, including antigen presentation, promotion of inflammation, immune regulation, and the maintenance of cellular homeostasis, among others. These findings highlight the robustness of utilizing WGCNA to identify immune cells with the strongest correlation to PCa lncRNA expression.

For instance, CD8+ T cells are more susceptible to apoptosis in terms of maintaining cellular homeostasis [12]. Follicular helper T cells (T follicular helper, Tfhs) can induce B cell activation and promote their differentiation into immunoglobulin-secreting cells [13,21]. The aberrant production of autoantibodies and B cell hyperactivation are important factors in the development of autoimmune diseases, such as Hashimoto’s thyroiditis, which is characterized by the accumulation of T and B lymphocytes in the thyroid and the production of autoantibodies targeting thyroid antigens [22]. In atherosclerotic plaques, the majority of T cells are T helper type 1 (Th1) cells [23]. Th1 cells secrete IFN-γ and TNF-α, which mediate macrophage activation [14].

Treg cells can regulate chronic inflammation, prevent tissue damage, suppress anti-tumor immune responses, and limit inflammation-associated tumor progression [15,24]. In the treatment of malignant tumors such as renal cell carcinoma and prostate cancer, the FDA-approved immune checkpoint inhibitors Ipilimumab and Tremelimumab, which target CTLA-4 (cytotoxic T-lymphocyte-associated protein 4), have demonstrated potent anti-tumor effects by targeting Treg cells [25].

During pregnancy, the depletion or dysregulation of myeloid-derived suppressor cells (MDSCs) is closely associated with pregnancy-related complications [16,17]. Preclinical studies have shown that treatment with dendritic cells (DCs) engineered to express the chemokine CCL21 can enhance the infiltration of DCs, CD4^+^, and CD8^+^ T cells into the lung tumor microenvironment (TME), thereby effectively reducing tumor burden [26,27]. Macrophages can differentiate into pro-inflammatory M1 or anti-inflammatory M2 phenotypes in response to different stimuli [28]. Under hypoxic conditions, mesenchymal-stem-cell-derived extracellular vesicles (MSC-EVs) promote lung cancer progression by upregulating miR-21-5p expression, inhibiting cell apoptosis, and inducing Macrophage polarization toward the M2 phenotype [29].

The correlation between immune cells and clinical features indicates that the infiltration levels of the seven immune cells increase with T stage, N stage, AGE, and Gleason score. Therefore, alterations in the proportions of these immune cells hold diagnostic potential for PCa. Although some immune cells in this study showed a low correlation with AGE (R < 0.2), indicating a weak relationship, most results were statistically significant (*p* < 0.05), suggesting the presence of potentially stable trends.

### 4.2. ceRNA Network

Emerging evidence suggests that ceRNA networks have significant implications in the initiation, progression, and prognosis of various cancers. The ceRNA regulatory network established in this study encompasses four lncRNAs (MEG3, PCAT1, SNHG19, and TRG-AS1), three miRNAs (hsa-miR-488-3p, hsa-miR-210-5p, and hsa-miR-137), and three shared mRNAs (H2AFJ, THBS1, and HPGD). These RNA molecules within the network exert crucial regulatory functions in different cancers, highlighting the predictive capability of our network. For instance, hydroxyprostaglandin dehydrogenase 15-(NAD) (HPGD) participates in the degradation and metabolism of Prostaglandin E_2_ (PGE_2_), which is implicated in inflammation response and tumor proliferation [30].

Histone 2A family member J (H2AFJ) is a member of the histone 2A family, which is a component of the nucleosome and plays a crucial role in gene regulation [31]. H2AFJ has oncogenic functions and is highly expressed in glioblastoma multiforme (GBM), particularly in mesenchymal GBM, where patients show poor response to temozolomide chemotherapy and have a worse prognosis [32]. Additionally, H2AFJ serves as a predictive factor for poor prognosis and resistance to neoadjuvant chemoradiotherapy in colorectal cancer patients [33]. Thrombospondin-1 (THBS1) is highly expressed in breast cancer [34] and melanoma [35], where it promotes tumor cell adhesion, proliferation, and apoptosis [36]. LncRNA PCAT1 promotes the proliferation of PCa cells and inhibits apoptosis [37]. The overexpression of PCAT1 enhances the proliferation and metastasis of non-small-cell lung cancer [38] and bladder cancer [39].

SNHG19 exerts its role in osteoarthritis by downregulating miR-34a through methylation, thereby inhibiting chondrocyte apoptosis [40]. Additionally, SNHG19 is aberrantly expressed in triple-negative breast cancer [41], pancreatic cancer [42], and other tumors. T cell receptor γ locus antisense RNA1 (TRG-AS1) upregulates the expression of WISP2 by competitively binding to miR-877-5p, thereby suppressing bone metastasis in breast cancer [43,44]. The overexpression of miR-181c-5p reverses the autophagy of HT22 cells induced by Maternally Expressed Gene 3 (MEG3) under hypoxic conditions and Oxygen and Glucose Deprivation/Reoxygenation (OGD/R) conditions [45]. Overexpressed MEG3 also promotes proliferation and inhibits oxidative stress, the secretion of inflammatory factors, and cell apoptosis, providing protection against P12 cell damage [46].

Has-miR-210 acts as a tumor suppressor, inhibiting cell proliferation and promoting apoptosis in ovarian cancer [47] and esophageal squamous cell carcinoma [48,49]. It also regulates HUVEC-mediated angiogenesis by modulating the NOTCH1 signaling pathway [50]. In an in vitro Alzheimer’s disease (AD) model using SH-SY5Y cells, SNHG19 regulates the toxicity induced by beta-amyloid (Aβ) accumulation through interaction with hsa-miR-137. The mRNA TNFAIP1 is likely a downstream target of the SNHG19/hsa-miR-137 axis in Aβ-treated SH-SY5Y cells [51].

The lncRNAs within the ceRNA network play significant roles in the regulation of gene expression. The core lncRNAs, miRNAs, and mRNAs within this network hold potential as novel indicators for the diagnosis and targeted therapy of prostate cancer (PCa). Furthermore, the interaction between SNHG19 and hsa-miR-137 in AD strengthens the reliability of our constructed ceRNA network.

### 4.3. mRNA Functional Enrichment

The shared mRNAs within the ceRNA network play crucial roles in PCa. GO enrichment analysis indicates that these mRNAs are significantly enriched in terms related to the response to testosterone (GO:0033574) and the response to progesterone (GO:0032570). Testosterone plays a vital role in the control and progression of PCa [52]. Testosterone treatment has been shown to improve the health status and quality of life of PCa patients with testosterone deficiency (TD) [53], and the endogenous serum testosterone level is not associated with the development of PCa [54].

Progesterone, a sex steroid hormone primarily synthesized in the corpus luteum, which is part of the ovary, with small amounts produced by the adrenal glands and testes [55], can regulate endoplasmic-reticulum-associated degradation (ERAD) and unfolded protein response (UPR) axes by mimicking androgen stimulation in PCa cells [56]. The enrichment of these shared mRNAs closely correlates with PCa, emphasizing their relevance to the disease.

Although this study provides an analysis of prostate cancer from the perspectives of the ceRNA network and immune microenvironment, identifying several potential key genes, there are still certain limitations. Due to experimental constraints, this study did not conduct in vitro functional validation and primarily relied on bioinformatics analysis using publicly available databases.

## 5. Conclusions

In conclusion, this study constructed a PCa ceRNA regulatory network encompassing 4 lncRNAs, 3 miRNAs, and 27 mRNAs, revealing the potential post-transcriptional regulatory mechanisms of non-coding RNAs in PCa. GO analysis showed that the genes in this network were significantly enriched in biological processes related to responses to hormones such as testosterone and progesterone, suggesting their key role in the hormone-dependent progression of PCa. Immune infiltration analysis revealed significant differences in tumor purity, estimate score, immune score, and stromal score between high and low ssGSEA score groups. Seven immune cell types showed specific distribution patterns across different T stages, N stages, and Gleason scores, implying their important role in the immune regulation of PCa. Therefore, this study provides insights into the molecular mechanisms of PCa from the perspectives of the ceRNA network and immune microenvironment, predicts potential prognostic biomarkers and immune therapy targets, and offers new approaches for immunotherapy.

## Figures and Tables

**Table 1:** Seed region binding between miRNAs and mRNAs.

3′UTR Binding Sites	Pairing With Target Gene	Site Type
Position 860-866 of THBS1 3′ UTR	5′GCUGCCUCCCAAAGGAGGGGCAG	7mer-A1
hsa-miR-210-5p	3′GUCACACGCCACCCGUCCCCGA	
Position 15-21 of HPGD 3′ UTR	5′NNACAGCUUAUGUGUUAGCCAUAG	7mer-A1
hsa-miR-210-5p	3′CUGGUAUGAUGCCUGUCGGUAA	
Position 15-21 of H2AFJ 3′ UTR	5′NNCCCUGACGCCGCCCUCAGGGAG	7mer-A1
hsa-miR-210-5p	3′GUCUUGUCCUGUCCAGUCCCG	
Position 308-314 of H2AFJ 3′ UTR	5′GGUCACCCUCGAGGCGUCAGGGG	7mer-m8
hsa-miR-210-5p	3′GUCUUGUCCUGUCCAGUCCCG	
Position 175412376-175412399 of HPGD 3′ UTR	5′AACUUUUAACAGUUACAGCAAUAA	8mer
hsa-miR-137	3′GAUGCGCAUAAGAAU-UCGUUAUU	
Position 175412827-175412834 of HPGD 3′ UTR	5′AUUCAAUAUUCUGCCUUUCAG	7mer-m8
hsa-miR-488-3p	3′CUGGUUCUUUAUCGGAAAGUU	
Position 14928678-14928683 of H2AFJ 3′ UTR	5′UCGGUUUGACUUUGCUUUCAA	7mer-A1
hsa-miR-488-3p	3′CUGGUUCUUUAUCGGAAAGUU	
Position 14928420-14928425 of H2AFJ 3′ UTR	5′UAGUAGCUGCUGUGCUUUCAU	6mer
hsa-miR-488-3p	3′CUGGUUCUUUAUCGGAAAGUU	
Position 39889031-39889036 of THBS1 3′ UTR	5′AUAUACACUUUUUUCUUUCAU	6mer
hsa-miR-488-3p	3′CUGGUUCUUUAUCGGAAAGUU	

Note: In the “Pairing with the target gene” column, blue bases represent the complementary pairing regions between the miRNAs and the 3′UTR sequences of the target mRNAs.

**Table 2:** Seed region binding between miRNAs and lncRNAs.

lncRNA/miRNA	Pairing with Target Gene	Site Type
MEG3	Target: 5′agucucugucuuuCCUUUCAc 3′	7mer-m8
hsa-miR-488-3p	miRNA: 3′cugguucuuuaucGGAAAGUu 5′	
TRG-AS1	Target: 5′aaguguGAAUUA-CCUUUCAu 3′	7mer-m8
hsa-miR-488-3p	miRNA: 3′cugguuCUUUAUCGGAAAGUu 5′	
SNHG19	Target: 5′cuauugaaGUGCUU-AGCAAUAa 3′	8mer
hsa-miR-137	miRNA: 3′gaugcgcaUAAGAAUUCGUUAUu 5′	

Note: In the “Pairing with the lncRNA” column, blue bases represent the complementary pairing regions between miRNAs and the 3′UTR sequences of the target lncRNA.

## Data Availability

The data of this study are from UCSC Xena, Ensembl, cBioportal, miRcode, ENCORI, and miRTarBasev databases; ethics committee approval was not required due to its exclusive use of public data. The dataset used during the current research period can be obtained from the following sources: The UCSC Xena database for miRNA data https://xenabrowser.net/datapages/?dataset=TCGAPRAD.mirna.tsv&amp;amp;amp;host=https%3A%2F%2Fgdc.xenahubs.net&amp;amp;amp;removeHub=https%3A%2F%2Fxena.treehouse.gi.ucsc.edu%3A443) (accessed on 30 March 2023). The Ensembl database for lncRNA data (https://ftp.ensembl.org/pub/release-109/gtf/homo_sapiens/) (accessed on 22 February 2023). Clinical data from the cBioportal database (https://www.cbioportal.org/study/clinicalData?id=prad_tcga) (accessed on 22 February 2023). The 28 immune cells obtained from the website (http://cis.hku.hk/TISIDB/data/download/CellReports.txt) (accessed on 21 April 2023). The data for constructing the ceRNA network is derived from the following databases: The miRcode databases (http://www.mircode.org/index.php) (accessed on 30 March 2023). The ENCORI databases (https://starbase.sysu.edu.cn/) (accessed on 1 January 2023). The miRTarBase databases (https://www.targetscan.org/vert_80/) (accessed on 30 March 2023). The inputs for both the miRcode database and miRTarBase database are miRNA IDs (Supplementary Table S2). Due to the periodic maintenance of the ENCORI database, which may result in temporary unavailability, we have uploaded the original data used in this database (Supplementary Materials).

## Supplementary Materials

The following supporting information can be downloaded at: https://www.mdpi.com/article/10.3390/genes16050527/s1, Figure S1: Relationships between seven immune cells and the tumor microenvironment. (A) Stromal Score. (B) Immune Score. (C) Estimate Score. (D) Tumor Purity; Table S1: 108 differentially expressed mRNAs in Pca; Table S2: 15 differentially expressed miRNAs in Pca; Table S3: 14 differentially expressed lncRNAs in Pca; Table S4: #the ssGSEA result file: ssgseaOut.txt; Table S5: #The gene sets for the 28 immune cell types.
