# Supplementary figures and images for: Competitive Endogenous RNA Network Involving Immune Subgroups, Infiltration, and lncRNAs in Prostate Cancer
Source: Genes (Basel). 2025 Apr 29;16(5):527. doi: 10.3390/genes16050527 (PMC12111210; doi:10.3390/genes16050527)

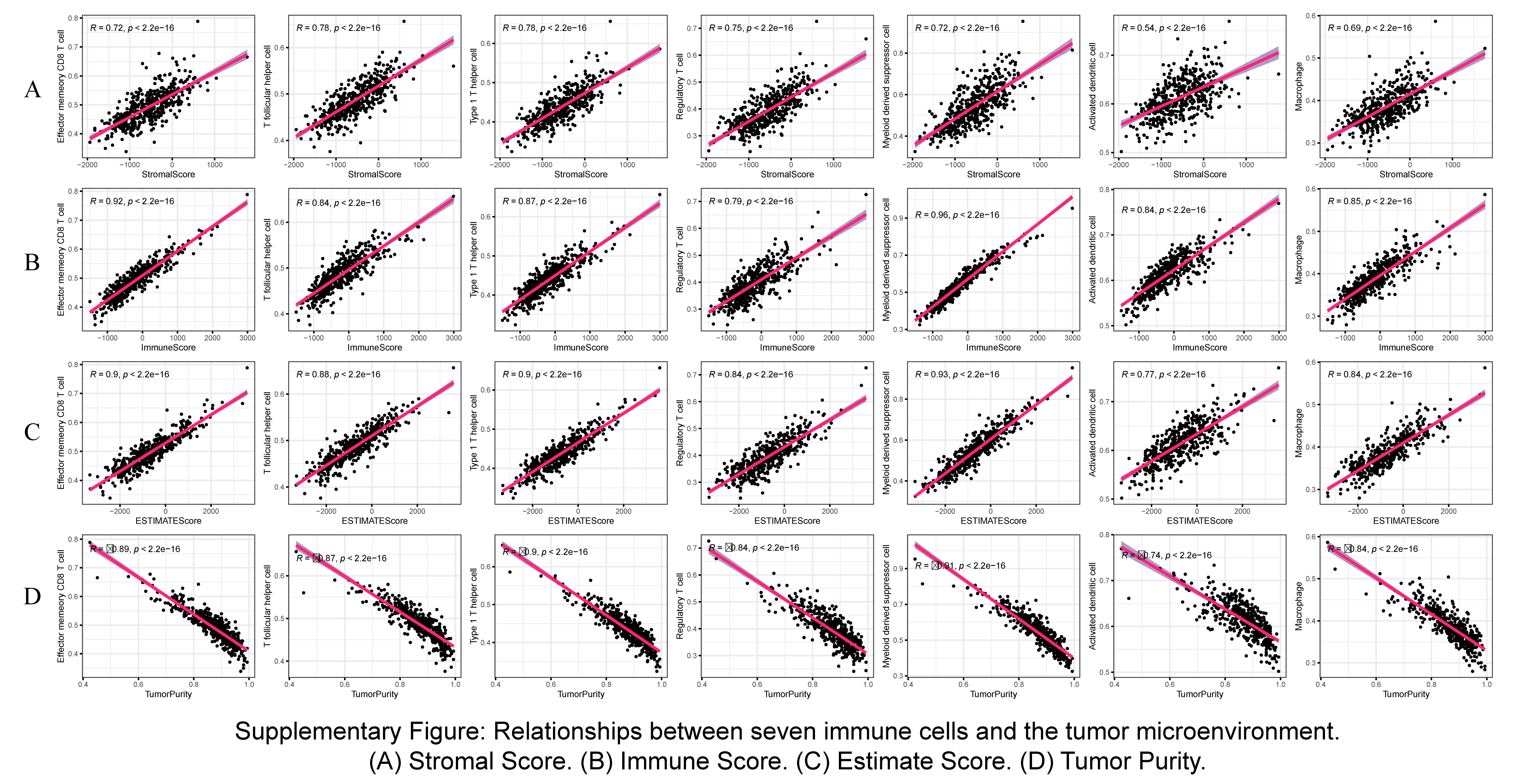

Supplement: Supplementary file 1 [file genes-16-00527-s001.zip › Supplementary Figure.tif]
